# Desexing Dogs: A Review of the Current Literature

**DOI:** 10.3390/ani9121086

**Published:** 2019-12-05

**Authors:** Silvan R. Urfer, Matt Kaeberlein

**Affiliations:** Dog Aging Project, Department of Pathology, University of Washington, Seattle, WA 98195, USA

**Keywords:** gonadectomy, desexing, orchiectomy, ovariectomy, ovariohysterectomy, spaying, neutering, dogs, epidemiology, lifespan, disease risk, population control, behavior, review

## Abstract

**Simple Summary:**

Desexing is a general term for interventions suppressing fertility in dogs, most commonly by surgically removing the testes or ovaries (“gonadectomy”). Desexing is promoted for population control, health benefits, and behavior modification. Surprisingly, the existing evidence shows no effect of desexing on population size in companion or shelter dogs; however, an effect has been shown for desexing female free-roaming dogs. Desexing has consistently been shown to change various health risks, including a reduction in pyometra and mammary tumor risk, as well as an increased risk of cranial cruciate ligament rupture, several forms of cancer, and obesity in both sexes. Other health effects vary considerably between breeds and sexes. A lifespan advantage in desexed dogs has consistently been shown in females, while the evidence is inconsistent in males, and the effect is smaller in studies that found one. There is more literature on behavioral effects in males than in females, and the evidence suggests reduced libido, roaming, conspecific mounting, and urinary marking in a large percentage of gonadectomized males, and reduced male dog aggression in a majority of males gonadectomized because of behavioral problems. The decision whether to desex dogs needs to be individualized based on the available evidence.

**Abstract:**

*Background*: Desexing dogs is promoted for population control, preventative healthcare, and behavior modification. Common methods are orchiectomy and ovariectomy/ovariohysterectomy. GnRH superagonist implants are available in some areas. Alternative methods like vasectomy and salpingectomy/hysterectomy are uncommon. The terminology used to describe desexing is inconsistent and contradictory, showing a need for the adaption of standardized terminology. *Population Control*: Surprisingly, empirical studies show no effects of desexing on population control in companion and shelter dogs despite desexing being consistently recommended in the literature. There is evidence for a population control effect in free-roaming dogs, where desexing also has benefits on zoonotic disease and bite risk. Population control in free-roaming dogs is mostly correlated with female, not male desexing. *Health and Lifespan*: Desexing affects numerous disease risks, but studies commonly neglect age at diagnosis and overall lifespan, age being by far the most important risk factor for most diseases. We argue that lifespan is a more important outcome than ultimate cause of death. A beneficial effect of desexing on lifespan is consistently demonstrated in females, while evidence for a beneficial effect in males is inconsistent. Studies are likely biased in desexing being a proxy for better care and desexed dogs having already lived to the age of desexing. Desexing reduces or eliminates common life-limiting diseases of the female reproductive system such as pyometra and mammary tumors, while no analogous effect exists in males. Disease risks increases across sexes and breeds include cruciate ligament rupture, various cancers, and obesity. Urinary incontinence risk is increased in females only. Various other disease risk changes show considerable variability between breeds and sexes. *Behavioral Effects*: Desexed males show reduced libido, roaming, conspecific mounting, and urinary marking frequency, as well as reduced male dog-directed aggression in a majority of males desexed for behavioral reasons. There is a detrimental effect on the risk and progression of age-related cognitive dysfunction. Desexed dogs may be less likely to cause bite injuries across sexes. The evidence for other effects such as human-directed aggression, human or object mounting, resource guarding, or shyness and anxiety is inconsistent and contradictory. There are few studies specific to females or individual breeds. *Conclusions*: The evidence for a beneficial effect of desexing is stronger in female than in male dogs; however, there is significant variation between breeds and sexes, and more research is needed to further elucidate these differences and to arrive at individualized evidence-based recommendations for clinical practice.

## 1. Introduction

Surgical desexing, usually by means of gonadectomy, is the most widely performed surgery in dogs and is commonly promoted as being part of responsible dog ownership in many English-speaking countries as well as parts of non-anglophone Western Europe. Desexing prevalence reaches 64% across both sexes in the United States [1], with some local jurisdictions having adopted mandatory desexing laws [2]. Desexing is somewhat less common in anglophone Western Europe, with prevalences reaching 54% in the UK [3] and 47% in Ireland [4]. In contrast, desexing is considered illegal without a clear medical indication in Germany [5] as well as some of the Nordic countries [6,7,8], with prevalences below 10% in Sweden [9]. 

A large variety of methods for desexing dogs has been described in the literature. While we attempt to give a complete overview of these methods, it is worth pointing out that gonadectomy is by far the most common form of desexing currently used in practice across the world, and the literature on its various consequences reflects this fact in that it predominantly focuses on the consequences of gonadectomy as opposed to other methods of desexing. Therefore, most of the evidence discussed in this review will necessarily be specific to gonadectomy as opposed to desexing in general.

Justifications for desexing put forward by the veterinary and shelter communities can generally be divided into three main lines of argument. These are (1) population control, (2) health benefits, and (3) behavioral benefits. For the purpose of this review, we will address all three of these aspects in separate sections.

## 2. Terminology

A wide range of terms has been used to describe the various methods of desexing, and no set of standardized terminology exists. For instance, both “neutering” and “castration” have been used by some authors as a sex-specific term for male gonadectomy, while others have used them as sex-neutral terms for gonadectomy in both sexes. “Sterilization” has been used as a sex-neutral term for gonadectomy, a sex-neutral term for methods that induce infertility without using gonadectomy, and a sex-specific term for female gonadectomy. In contrast, “spaying” is consistently used as a sex-specific term for female gonadectomy; however, it may or may not also indicate concurrent hysterectomy and has occasionally also been used to denote hysterectomy without ovariectomy or with an ovarian autograft (“ovary-sparing spay”).

In the interest of consistency and clarity, for the scope of this review we will use “desexing” as a sex-neutral term for any procedure intended to cause temporary or permanent loss of fertility, “gonadectomy” as a sex-neutral term for the surgical removal of the gonads in both sexes, “orchiectomy” as a sex-specific term for gonadectomy in males, “ovariectomy” as a sex-specific term for gonadectomy in females, and “ovariohysterectomy” as a sex-specific term for gonadectomy combined with hysterectomy in females. Specific methods not involving orchiectomy or ovariectomy will be referred to using their commonly used names as taken from the literature.

## 3. Desexing Methods

In the broadest sense, desexing methods can be divided into surgical and non-surgical procedures [10,11,12]. Given the significant anatomical and physiological differences between male and female dogs, we will further distinguish between methods used in males and methods used in females as appropriate.

### 3.1. Surgical Methods

Surgical methods are the most widely used form of desexing in dogs, and are the most commonly performed surgeries in the species overall [7,10,13,14,15,16]. They can be differentiated into surgeries that remove the gonads (orchiectomy, ovariectomy, ovariohysterectomy) and surgeries that do not (vasectomy, salpingectomy, hysterectomy), the former methods being considerably more common than the latter.

#### 3.1.1. Orchiectomy

Orchiectomy (also called orchidectomy) is the surgical removal of the testes in male dogs. Two main surgical methods exist to achieve this goal, which are referred to as the open and the closed technique. In both techniques, the patient is placed in dorsal recumbency and the surgeon places a skin incision cranially from the scrotum through which the testicles are extracted. In the open technique, the surgeon then also opens the vaginal tunic before placing a ligature around the vas deferens and testicular artery and removing the testicle; in the closed technique, the vaginal tunic is left in place and a ligature is placed around the tunic as well as the testicular artery, cremaster muscle, and vas deferens inside the tunic before removing the testicle and surrounding structures distal of the ligature. In addition to these two primary methods, a sutureless scrotal approach has recently been described for use in juvenile male dogs [17], and testicular implants for orchiectomized dogs are also commercially marketed [18].

Short-term complications of orchiectomy are rare and are mostly limited to the usual non-specific surgical risks, i.e., anesthetic complications, swelling, hematoma, bleeding, and infection [10]. 

#### 3.1.2. Alternative Surgical Methods in Males

Various surgical desexing methods not involving orchiectomy have been proposed for male dogs; however, none of them have been widely adopted in practice. The most common such surgery is vasectomy, which consists of the surgical transection of the vas deferens, leading to azoospermia and thus, infertility without affecting testosterone levels [19]. Methods include open [20,21] and endoscopic vasectomy [22], as well as experimental methods including implantation of an intravasal filtering device [23], and non-scalpel methods involving ultrasound [24], laser surgery [25,26], and electrocoagulation of the vas deferens [27]. 

Vasectomy does not result in changes in circulating testosterone levels, libido, or other male-specific behaviors, and as such has been suggested for use in free-roaming and/or feral dogs as a potentially more effective means of population control than orchiectomy [28,29,30]. Specific complications of vasectomy appear to be rare in dogs and can include spermatocele [31], sperm granuloma [31,32], and testicular degeneration [33].

In addition to the various forms of vasectomy listed above, surgical methods described in the literature also include the Burdizzo castrator [34], while use of the elastrator is considered inhumane in dogs [35]. 

#### 3.1.3. Ovariectomy and Ovariohysterectomy

Ovariectomy (also called oophorectomy) is the surgical removal of the ovaries in female dogs, while ovariohysterectomy refers to the surgical removal of both the ovaries and the uterus [10]. Ovariohysterectomy is generally considered the method of choice in older females. In North America, it is also the most commonly used desexing procedure in young females, while ovariectomy without concurrent hysterectomy is generally considered the method of choice for desexing young females in Western Europe [36,37].

In contrast with orchiectomy, both ovariectomy and ovariohysterectomy require a laparotomy in order to access the surgical site. There are two commonly used surgical approaches for both procedures in female dogs, which are the midline incision and the flank incision. The midline incision is the more commonly used approach of the two, while the flank incision is mostly performed in shelter medicine settings, in small or narrow-bodied dogs, or in dogs with significant mammary development [37,38]. 

The midline incision is performed with the patient in dorsal recumbency and is placed in the linea alba slightly caudal of the umbilicus. The flank incision is performed with the patient in lateral recumbency, with the right flank approach being more commonly used due to anatomical considerations [38]. The incision site lies caudal of the midpoint between the iliac crest and the last rib, with the abdominal muscle being dissected bluntly. Both methods then use isolation and ligation of the ovarian pedicle and removal of the ovaries, followed by ligature of the uterine body and cervix and removal of the uterus in case of ovariohysterectomy.

Laparoscopic ovariectomy and/or ovariohysterectomy techniques (sometimes including electrocoagulation) have also been described for female dogs, and may be associated with fewer signs of post-operative pain and faster recovery times; however, they are also associated with higher cost [39,40,41].

As is the case in orchiectomy, short-term complications of ovariectomy and ovariohysterectomy are rare and mostly limited to the usual non-specific surgical and anesthetic risks as listed above [10].

#### 3.1.4. Alternative Surgical Methods in Females

Alternative surgical desexing methods in female dogs are not widely used in practice and include salpingectomy, i.e., the surgical transection of the fallopian tubes [42], hysterectomy without ovariectomy, and ovariectomy or ovariohysterectomy combined with an ovarian autograft [43,44]. Their medical consequences are not well studied. As is the case in vasectomy, these methods do not change the hormonal status of the dog, meaning that the health consequences can be expected to be similar to these found in intact individuals inasmuch as the susceptible organs remain in the body.

### 3.2. Non-Surgical Methods

Various non-surgical methods to induce infertility and/or suppress libido and other hormone-mediated aspects of behavior in dogs have been described in the literature. They can roughly be divided into hormonal methods, immunological methods, and chemical or physical sclerotization methods [11]:

#### 3.2.1. Hormonal Methods

The most commonly used hormonal methods have used progestins to induce reversible infertility in both male and female dogs and to also suppress both libido and estrus, as well as other specific effects of testosterone and estrogens. They include competitive steroid hormone inhibitors such as cyproterone acetate [45], as well as progestogens such as megestrol acetate, medroxyprogesterone, and proligestone [46]. Suppression of estrus and fertility is readily achieved in female dogs using these methods; however, they have been shown to be less reliable in achieving infertility in male dogs [47]. Exogenous testosterone has also been used to successfully suppress fertility in both male and female dogs; however, its undesirable androgenic side effects render it impractical for clinical use [47]. 

More recently, GnRH superagonists such as deslorelin applied in the form of slow-release subcutaneous implants have been used to successfully induce reversible infertility lasting several months to years in both male and female dogs. After an initial gonadotropin surge that in females may induce fertile estrus, these implants suppress the release of gonadotropins in the pituitary gland, which results in downregulation of the gonads mimicking the effects of gonadectomy, and resulting in complete infertility and undetectable levels of sex steroids within 6–8 weeks after implantation [48,49,50]. While these implants are not currently commercially available in North America, they are now widely used in veterinary practice in both Europe and Australia. Their use has also been suggested to delay the onset of puberty in both male and female dogs by several years [51,52,53].

Phytoestrogens have also been suggested as a possible method of inducing infertility in male dogs; however, this has not been applied in clinical practice thus far [54]. 

#### 3.2.2. Immunological Methods

There have been several immunological methods investigated to achieve infertility in both male and female dogs; however, while there have been some promising results in studies on free-roaming dogs [55,56], none of the studied products have thus far been adopted in clinical practice. Proposed and tested targets for immunization have included sperm antigens, the zona pellucida of the oocyte, GnRH, and LH [56,57,58,59,60,61,62]. 

#### 3.2.3. Sclerotization Methods

Sclerotization methods have thus far only been used on male dogs, have used both chemical and physical means of achieving sclerotization, and have been aimed at either the testicle, the epididymis, or the vas deferens. 

Various chemical sclerotizing agents have been proposed, among them intratesticular injections of calcium chloride or zinc gluconate [63,64,65], intraepididymal injections of chlorhexidine gluconate, zinc arginine, or formalin [28,66,67], and injections of ethanol, silver nitrate, acetic acid, formalin, sodium tetradecyl sulfate, sodium morrhuate, or potassium permanganate into the vas deferens [68]. Among these methods, intratesticular injections of calcium chloride or zinc gluconate have seen limited use in private veterinary practice and particularly in shelter medicine.

Physical sclerotization has used ultrasound application aimed at either the whole testicle [69,70] or the epididymis [24], but has not been used in clinical practice thus far. 

## 4. Population Control

Starting in North America during the 1970s, concerns about pet overpopulation and implementing population control measures have historically been at the forefront of desexing advocacy originating from both the veterinary profession and the shelter community, focusing on both animal welfare and public health aspects associated with free-roaming dogs [71,72,73,74,75]. However, considering both the substantial body of literature advocating in favor of desexing as a means of population control and the widespread use of desexing in shelters and by private practice veterinarians over the last 40–50 years [3,13,14,76,77,78,79], the body of evidence investigating the effectiveness of desexing to actually achieve population control in companion and shelter dogs is surprisingly slim [80,81], and the evidence from such studies does not generally support the existence of an effect of desexing programs on population control, as discussed below. 

Another concern in both desexing advocacy and the study of its effects on population control is a lack of sex-specific considerations. In mammalian population dynamics, it is generally accepted that reproductive rates in a population predominantly depend on the availability of fertile females, while the number of fertile males does not directly affect population size at the desexing rates that are achievable in practice, indicating that inhibiting female reproduction is the intervention that will predominantly determine outcomes of desexing interventions [55,82,83,84]. However, these considerations have not been incorporated into the currently recommended protocols [13,77,79] and are largely absent from the veterinary literature. 

In summary, the available empirical evidence does not support the notion that gonadectomy programs have an effect on population size in companion and shelter dog populations, while there is evidence for such an effect in free-roaming dog populations. Demographic surveys suggest that this lack of an effect in companion and shelter dog populations may in part be due to the fact that at least in the US environment, roughly two thirds of dog litters are intentionally bred rather than accidental [85]. There is also evidence that dogs being relinquished to shelters may be more strongly correlated with the existence of behavior problems than with population size. In addition, the lack of a specific focus on females that is common in companion dog gonadectomy programs may also contribute to the lack of an effect on population control.

The existing literature on the effects of desexing on population control in dogs can be divided into studies based on mathematical modelling, empirical studies on companion and shelter dog populations, and empirical studies on free-roaming dog populations.

### 4.1. Studies Using Mathematical Modelling

Mathematical modelling studies use various assumptions about a modelled dog population based on empirical surveys of existing dog populations, and then use these assumptions to test the influence of various variables and interventions on birth rates, mortality rates, as well as immigration from and emigration into adjacent populations in order to estimate the impact on population dynamics and ultimately population size over various lengths of time. 

Such studies have thus far mostly been limited to scenarios using free-roaming dogs, or scenarios where a significant number of such dogs exist alongside the population of owned companion dogs. The most important factors identified using such studies are generally the percentage of fertile females [82,83], the carrying capacity of the environment [83,86,87], a sustained desexing effort over time as opposed to a one-time intervention [83,86], and the introduction of intact animals from adjacent populations and/or through animal abandonment [86].

### 4.2. Empirical Studies on Companion and Shelter Dogs

Empirical studies on the effects of gonadectomy on companion and shelter dog population dynamics exist from the United States, Canada, and Brazil. These studies generally investigate populations over various points in time before and after an intervention was implemented, or compare areas where an intervention was implemented to similar areas without such an intervention. Methods used include owner surveys as well as records taken from private and public animal shelters, and the variables analyzed generally include shelter intake and euthanasia rates over time. Only one study published thus far has considered existing population dynamics trends preceding the time at which the intervention was introduced [88]. 

All studies in companion and shelter dogs thus far have examined the impact of gonadectomy across both sexes and have not considered sex-specific effects. With one exception, the available empirical studies have not found an effect of gonadectomy programs on common measures of population size in companion or shelter dogs.

Analysis of data generated from the “Maddie’s Fund” initiative promoting low-cost gonadectomy programs in the US States of Utah and Alabama, as well as in Maricopa County, AZ, Alachua County, FL, and the city of Lodi, CA found that these programs significantly increased the percentage of gonadectomized dogs in the population, but did not affect shelter intake rates [81].

A study in two populations in New Hampshire and Austin, TX studied shelter intake and euthanasia numbers before and after the implementation of a gonadectomy intervention; in New Hampshire, following the introduction of a “neutering initiative” promoting low-cost gonadectomy in both sexes, a beneficial effect was found in cats, but not in dogs. In Austin, a free gonadectomy program was introduced in specific areas of the city and was shown to slow the increase of dog intake and euthanasia rates in the targeted areas as compared to areas where no such intervention had taken place [89].

A study in Transylvania County, NC, examined the impact of a subsidized gonadectomy clinic on shelter intakes and euthanasia before and after the clinic opened. In this study, the shelter intake rate had already been decreasing before the clinic had opened, and the decrease did not accelerate afterwards. Euthanasia rates had also been decreasing before the clinic had opened; however, they stabilized afterwards [88].

A study in Santa Clara and San Mateo Counties in California found that the introduction of gonadectomy vouchers by the shelter system resulted in a reduction in animal shelter intake numbers in cats, but not in dogs [90].

In one study, a door-to-door outreach campaign promoting gonadectomy in an underserved area of New York City had no significant effect on the rates of animal abandonment compared to a control population [91]. 

In a study of two Native American communities in North Saskatchewan, Canada, consisting of an environment where both owned companion dogs and unowned free-roaming dogs were present, introducing a subsidized gonadectomy program for companion dogs combined with capture and gonadectomy of free-roaming dogs resulted in a significant increase in the percentage of gonadectomized dogs, a decrease in the number of litters observed, and a decreased population size nine months after the intervention; however, the decrease in population size was caused by placing unwanted dogs that were identified during the intervention with households outside the communities [92].

Finally, in one study in the Brazilian city of Curitiba, it was found that a three-year population management and control program promoting gonadectomy to companion dog owners did not significantly affect the number of dogs in the city [93]. 

### 4.3. Empirical Studies on Free-Roaming Dogs

Empirical studies on free-roaming dogs have largely been conducted in environments where dogs reproduce outside of human interference and where the presence of free-roaming dogs poses significant risks to human health, either as disease reservoirs for infections such as rabies [56,94,95], or by causing a significant number of bite injuries [96]. In these situations, free-roaming dogs are generally trapped, desexed using gonadectomy or non-surgical methods, vaccinated against rabies and other diseases, treated against infections and common parasites, and then released [56,94,95,96,97,98]. 

In one study in Jaipur, India conducted between 1994 and 2002, it was estimated that 65% of all female dogs were gonadectomized during this period, which resulted in a decrease in the free-roaming dog population size of 28% over the period [94]. Another study conducted before and after implementation of a gonadectomy/rabies vaccination program in Jodhpur, India found a significant decline in the dog population size in three out of five surveyed areas following implementation of the program. A different study conducted in several Indian cities in 2011 found that gonadectomy also resulted in improved body condition scores and reduced disease and parasite load (other than ticks) in gonadectomized dogs as compared to intact controls [97].

In this context, it has also been suggested that vasectomizing rather than gonadectomizing male dogs may exert a beneficial effect in that these infertile males will continue to compete with fertile males for the available fertile females, thus reducing the rates of successful impregnation in the latter [30]. To our knowledge, this hypothesis has not been tested in practice thus far.

## 5. Lifespan and Disease Prevalence

### 5.1. Lifespan

As we summarize below, much has been published on various disease risk changes associated with desexing or not desexing; however, most of these studies did not take lifespan or age at diagnosis into account. This is problematic for two reasons: First, age is by far the most important risk factor for a wide variety of diseases in dogs, including many of the diseases for which a correlation with desexing has been described [99,100,101,102]. A change in individual disease prevalence may therefore simply indicate a difference in lifespan. Second, while desexing or not desexing will indeed change a dog’s risk of developing various life-limiting diseases, it ultimately does not prevent death. This may seem obvious and even trivial when stated in this manner; however, a large part of the literature appears to neglect this fact and focuses largely on causes of death. Given that lifespan is finite in both intact and desexed dogs, we argue that more emphasis should be placed on lifespan (and, ideally, healthspan) as a relevant measure of positive or negative health outcomes associated with desexing dogs, regardless of ultimate cause of death.

While studies on individual disease risk changes associated with desexing are abundant, the number of studies examining the effects of desexing on lifespan is more limited [103,104,105,106,107,108]. Studying the effects of desexing on lifespan also adds some confounders in that at least in the Western context, desexing is often a proxy for overall better veterinary care, which may result in an overestimation of any beneficial effect of desexing on lifespan. In addition, any desexed dog must necessarily already have lived to the age at which it was desexed, which skews the desexed population towards older dogs by removing any dog that died before the age at which desexing would have been carried out [107]. Furthermore, the presence of right censored data may further skew the results of lifespan studies based exclusively on death records by resulting in underestimated lifespans that may not be consistent between the groups considered [109].

The first study including systematic information on lifespan and desexing was published in 1982 based on necropsy data from n = 2002 dogs from a private veterinary hospital in New England covering necropsies performed between 1962 and 1976 [110]. This study found a significant lifespan advantage of gonadectomized over intact dogs in both sexes, with intact females living to a mean age of 4.7 years, while gonadectomized females lived to a mean age of 8.6 years. In the same study, intact males lived to a mean age of 4.8 years, while gonadectomized males lived to a mean age of 9.9 years. When considering only dogs that had lived to at least 2 years of age, this was 7.7 vs. 8.8 years in intact vs. gonadectomized females, and 8.0 vs. 9.9 years in intact vs. gonadectomized males. Interestingly, this study did not find a body size effect on lifespan, which contradicts much of the subsequent literature on body size and lifespan in dogs.

Another study investigated lifespan in dogs in the UK based on owner surveys representing n = 3121 deceased dogs, and found that intact females had died at a median age of 12 years, while gonadectomized females had died at a median age of 10 years, 10 months. In the same study, intact males had died at a median age of 10 years, 11 months, while gonadectomized males had died at a median age of 10 years, 8 months. The authors reported that desexed females lived significantly longer than intact females as well as males regardless of desexing; however, there was no significant difference in lifespan between desexed and intact males [103].

In 2013, a study based on the Veterinary Medical Database (VMDB), which aggregates data on deceased dogs from US veterinary teaching hospitals, found that based on data covering the years 1984–2004 from n = 70,574 deceased dogs, desexing increased male life expectancy by 13.8%, while it increased female life expectancy by 26.3% [108]. Additionally, in a later analysis based on the same VMDB data and this time including juvenile dogs and dogs of unknown desexing status (n = 80,958), it was found that intact males lived significantly longer that intact females, but gonadectomized females were the longest living demographic. The same study also analyzed data from the VetCompass database of private veterinary practices in the UK spanning the time period 2009–2011 (n = 5095 deceased dogs) and came to the same conclusion [105].

A breed-specific study based on n = 652 deceased Golden Retrievers with known ages at death necropsied at a US veterinary teaching hospital between 1989 and 2016 found that gonadectomized females lived significantly longer than intact females; however, there was no significant lifespan advantage of gonadectomized over intact males [111]. In contrast, an international study based on an online survey of n = 2505 Vizslas born between 1992 and 2008 found no difference in lifespan between intact and gonadectomized dogs [112].

Most recently, data based on the Banfield network of primary care veterinary clinics in the contiguous US including ages at most recent visit in addition to known death data (n = 2,504,518) found that intact females lived to a median age of 13.77 years; gonadectomized females, 14.35 years; intact males, 14.09 years; and gonadectomized males, 14.15 years [107].

In summary, all large-scale studies of the effects of desexing on lifespan are based on data from either the US or the UK, all of them investigate gonadectomy as opposed to other methods of desexing, all but one exclusively rely on data from deceased dogs, and none of them take age at desexing into account. Additionally, the available studies consistently find a survival advantage in gonadectomized over intact females, while the data in males are inconsistent, and where such an effect is described in males, it is consistently of lesser magnitude than is the case in females. This is potentially problematic when considering desexing across both sexes as part of a study, as the stronger effect in females may overshadow the weaker or non-existent effect in males [107].

The issue of age at desexing in lifespan studies is an important question that has not been addressed in any large-scale studies thus far. In this context, it is likely that the amount of time during which a dog is exposed to sex hormones influences its disease risks and overall life course [112,113]. 

### 5.2. Individual Disease Risks

#### 5.2.1. Existing Review Articles

A large body of literature exists covering various long-term health aspects of desexing, with most papers focusing on individual disease and/or cause of death risks; however, many of the existing studies on individual disease risks are limited to one breed or to a relatively low number of breeds [104,112,114,115,116,117,118,119,120].

Various general review papers giving overviews of the reported disease risk changes associated with desexing or not desexing have been published at this point [15,78,121,122], including several reviews specifically focusing on the long-term health effects of pediatric desexing [16,123,124,125,126]. In addition, several disease-specific review articles exist examining the influence of desexing on cancer development [127], mammary tumor risk [128], pyometra [129], and urinary incontinence in female dogs [130]. 

As explained above, our view is that individual disease risks are considerably less important than lifespan when measuring health outcomes of desexing in dogs, and given the extensive amount of existing review articles including articles on individual disease risks, we have aimed to keep this part of the present review fairly concise. Based on the extent of the available disease-specific literature, we will focus on diseases of the reproductive system *sensu lato*, tumors not involving the reproductive system, joint disorders, urinary tract disease, and weight gain and obesity.

#### 5.2.2. Diseases of the Reproductive System

Seeing as gonadectomy involves removal of the gonads, it eliminates the risk of any diseases directly affecting these organs. In males, this includes the various forms of testicular tumors, which consist of Leydig cell tumors, Sertoli cell tumors, interstitial cell tumors, seminomas, and teratomas [131,132,133,134,135,136,137,138,139,140]. Testicular tumors predominantly occur in aged dogs, and more than half of all testicular tumors occur in cryptorchid dogs [131]. A breed predisposition has been described in Maltese, which may be linked to an increased risk of cryptorchism in the breed [131]. In females, the ovarian tumors described in the literature include epithelial tumors, granulosa cell tumors, Sertoli–Leydig cell neoplasms, sex cord stromal tumors, and germ cell tumors including teratomas [141,142,143,144,145].

In females, ovariohysterectomy includes the removal of the uterus, eliminating the risk of various uterine diseases, including pyometra. In a study of over 200,000 insured dogs in Sweden, the 12-month pyometra risk in intact female dogs aged under 10 years was 2%. This study found breed predispositions in Rough Collies, Rottweilers, Cavalier King Charles Spaniels, Golden Retrievers, Bernese Mountain Dogs, and English Cocker Spaniels, while breeds with below-average risk were Drevers, German Shepherds, Dachshunds, and Swedish Hounds [146]. A similar study on 260,000 insured Swedish dogs found that the mean age of diagnosis was 8 years, and that 19% of all intact females had been affected with pyometra by age 10 [6].

Gonadectomy in female dogs is commonly thought to result in a significant reduction in mammary tumor risk, especially if performed prior to the first estrous cycle. Mammary tumors are some of the most commonly diagnosed tumors in intact female dogs, with roughly 90% of them being epithelial in nature, with the remaining 10% being sarcomas and mixed type tumors [147]. Roughly 50% of mammary tumors in female dogs are benign, and 50% of the malignant tumors lead to metastasis [148]. In the largest study thus far, 13% of 260,000 insured intact female dogs had developed mammary tumors by age 10 [6]. 

There are multiple studies supporting the view that gonadectomy (particularly when done early in life) has a protective effect against mammary tumors in female dogs [120,149,150,151]; however, a recent review of the literature based on Cochrane guidelines judged that the existing evidence in favor of this view is weak overall [128].

In males, desexing eliminates the risk of benign prostatic hyperplasia, which can be found in more than 95% of aged intact male dogs. However, it only causes clinical signs in a relatively small subset of these dogs, and curative treatments are available medically or by desexing [152,153,154,155,156]. Desexing also decreases the risk of perianal adenomas in male dogs [157]. Surprisingly, desexing increases the risk of malignant prostate tumors in dogs, with gonadectomized dogs having 3.9 times the risk of intact ones in a study on 70 dogs. Most of these prostatic neoplasms in dogs were carcinomas of ductal origin and were not androgen-dependent [158]. However, this is a relatively rare disease, which limits its importance in the decision making process in male dogs [122].

Finally, desexing has been found to decrease the prevalence of transmissible venereal tumor across both sexes in areas where the disease is enzootic, presumably by reducing the number of sexual encounters in the population associated with the loss of libido in desexed dogs [159].

In summary, the evidence suggests a beneficial effect of desexing on the occurrence of common and commonly life-limiting diseases of the reproductive system in female dogs, particularly concerning pyometra and (to a somewhat lesser degree) mammary tumors, with large variance in breed-specific pyometra risks. In males, the beneficial effects of desexing are most dramatic on benign prostatic hyperplasia, which is a common, but not generally life-limiting disease, while the influence of male desexing on life-limiting diseases is mixed and limited to comparably rare diseases.

#### 5.2.3. Tumors not Involving the Reproductive System

Desexing has been shown to increase the risk of several non-reproductive tumors in dogs. These risk increases can affect both sexes or may only be present in females, depending on the tumor. In contrast, apart from perianal adenomas in male dogs mentioned under the previous point, there are no non-reproductive tumors that show a risk decrease following desexing in dogs, and no non-reproductive tumor risk increases that are specific to desexed male dogs [127]. 

Non-reproductive tumors with an increased risk across both sexes following desexing are cardiac and splenic hemangiosarcoma [112,117,160,161,162], appendicular osteosarcoma [162,163,164,165], lymphoma [112,117,162,166], and transitional cell tumors of the bladder [167,168]. An increased risk for mast cell tumors has been described that may be specific to female dogs [120,162].

There have been a few breed-specific studies on gonadectomy and tumor risk. In a study on Golden Retrievers based on dogs necropsied at a US veterinary teaching hospital between 1989 and 2016, it was found that a greater proportion of gonadectomized females died of cancer than was the case in intact females; however, gonadectomized females also lived longer, and unsurprisingly, age was found to be a more important factor for cancer risk than gonadectomy. In the same study, gonadectomized male dogs did not live longer than intact males, and there was no difference in tumor related mortality [111]. A study based on patient records of n = 1015 Golden and n = 1500 Labrador Retrievers from the same US veterinary teaching hospital between 2000 and 2012 found that the increase in tumor risk following gonadectomy was strongest in female Golden Retrievers, and that the tumor risk increases associated with gonadectomy were stronger in this breed as compared to Labrador Retrievers [114]. Additionally, a study of n = 1170 German Shepherds from the patient records of the same veterinary teaching hospital collected between 2000 and 2014 found no difference in cancer risk in gonadectomized over intact males, and with the exception of mammary tumors, there was no increased cancer risk in gonadectomized over intact females [120].

In a cohort of n = 2505 Vizslas born between 1992 and 2008 that was studied through a worldwide online survey, it was found that gonadectomy increased tumor risk across both sexes. In this study, mast cell tumors were diagnosed at significantly earlier ages in gonadectomized as opposed to intact dogs, while there was no difference in ages at diagnosis in lymphoma and hemangiosarcoma. Other cancers were also diagnosed at significantly earlier ages in gonadectomized dogs as compared to intact ones across both sexes. This study found no difference in lifespan between intact and gonadectomized dogs overall [112]. 

In summary, there is consistent evidence for an increased osteosarcoma, lymphoma, transitional cell cancer, and hemangiosarcoma risk in gonadectomized dogs of both sexes, while the evidence is inconsistent for other types of tumors and suggests that there may be considerable variation between breeds and sexes. Additionally, and as mentioned above, the most important risk factor for tumor development is age [99,100,101], and many of the present studies on tumor risk do not take age into account, meaning that some of these findings may also reflect a difference in lifespan as opposed to a direct influence of desexing on tumor development. Unsurprisingly, when studies take age into account, it is shown to be a more important tumor risk factor than gonadectomy [111]. In addition, many studies are limited to one or a relatively low number of dog breeds, which may represent breed-specific risk changes and therefore raise concerns regarding generalizations to the whole dog population. 

#### 5.2.4. Joint Disorders

Desexing has been shown to affect the risk for various joint disorders, both across the general dog population and in individual breeds. Disorders that have been reported as being more common in desexed dogs include craniate cruciate ligament (CCL) injury, hip dysplasia (HD), elbow dysplasia (ED), and osteoarthritis in general. The most consistently reported joint disorder with an increased risk in gonadectomized dogs of both sexes is CCL injury, which has been reported both in the general population and in individual breeds such as German Shepherds as well as Golden and Labrador Retrievers [114,116,117,120,162,169,170], and with some of the available studies reporting an increased risk in dogs that were desexed before puberty. 

Other joint disorders that have been found to occur more commonly in desexed dogs are HD in gonadectomized male Golden Retrievers [117]; overall joint disorder risk (HD, ED, CCL rupture) in gonadectomized German Shepherds of both sexes [120], Golden Retrievers of both sexes [114], and female Labrador Retrievers [114]; ED and CCL rupture in male Labrador Retrievers that were gonadectomized before six months of age [114]; and CCL injury and osteoarthritis in Golden Retrievers of both sexes that were gonadectomized before six months of age [116].

While one study has described a decrease in bone density in ovariectomized laboratory Beagles [171], this result has not been reproduced in a later study [172], and to the authors’ knowledge, the veterinary literature does not contain any indications that ovariectomy is a risk factor for clinically relevant bone density loss in female dogs.

In summary, the risk of CCL injury appears to consistently increase with desexing across the general dog population as well as in the individual breeds studied, while there appears to be considerable variability in HD, ED and osteoarthritis risk increase depending on breed, sex, and age at desexing. More research is needed particularly in the fields of breed- or size-specific ED and HD risk, as well as age at desexing.

#### 5.2.5. Urinary Disease

There is evidence from multiple studies that gonadectomy increases the risk of urinary incontinence in female dogs, and that this risk increase is also associated with larger body size [120,125,173,174,175,176,177,178], with a prevalence of urinary incontinence of up to 20% of gonadectomized females reported in the literature [179]. This appears to be a sex-specific effect, as orchiectomy does not appear to be associated with an increased incontinence risk in male dogs [180]. However, a 2012 systematic review of the literature based on Cochrane criteria concluded that the evidence for an increased risk of urinary incontinence in gonadectomized female dogs was weak, and most consistent in female dogs that had undergone the procedure before three months of age [130]. Apart from urinary incontinence, gonadectomized female, but not male dogs have also been reported to be at a higher risk of struvite urolithiasis as compared to intact females and intact males respectively [181].

Interestingly, it has been reported that urinary incontinence in gonadectomized female dogs can be successfully treated using GnRH superagonist implants, which as we report under point 3.2 are an increasingly common means of desexing in Western Europe and Australia [179,182]. These implants work by suppressing the release of gonadotropins, while surgical gonadectomy increases the release of gonadotropins due to the removal of the negative feedback mechanism provided by the presence of sex steroids secreted in the ovaries. This in turn would indicate that the urinary incontinence risk increase seen in gonadectomized female dogs is not caused by the lack of sex steroids associated with desexing, but rather by the increased levels of gonadotropins induced by ovariectomy.

In summary, the effects of desexing on urinary incontinence and urolithiasis risk increase appear to be limited to female dogs. There is a substantial amount of evidence for ovariectomy and ovariohysterectomy being associated with an increased risk of urinary incontinence, particularly in large dogs that have been desexed at an early age. This effect may be specific to desexing methods involving ovariectomy and may not be associated with methods that do not affect the release of gonadotropins.

#### 5.2.6. Weight Gain and Obesity

There are multiple studies from a wide variety of geographic areas linking desexing to an increased risk of obesity in dogs. These studies include dog populations from the US, the UK, Continental Europe, Australia, China, and Japan [116,183,184,185,186,187,188,189,190,191]. While one recent study performed in Danish dogs found an increased obesity risk associated with desexing that was limited to male dogs [189], most other available studies find that the effect is present in both sexes. In addition, an international study of 926 overweight dogs undergoing a weight loss diet for three months found that intact dogs of both sexes lost significantly more weight during the study period than desexed dogs [192]. 

One US study comparing patient populations between a low-cost and a general practice veterinary clinic found that obesity in dogs was not associated with socioeconomic status of the owners, but that desexed dogs were consistently more likely to be obese across both populations [183]. In addition, diabetes mellitus has been reported to be more common in neutered male dogs as compared to intact males, and obesity is a known risk factor for diabetes mellitus in dogs [193]. Interestingly, in one study that measured food intake in free-fed intact female dogs across their sexual cycle, it was found that caloric intake was lowest during estrus and highest during anestrus, and that caloric intake increased after gonadectomy, indicating that the presence of female sex steroids may have a negative effect on caloric intake [194].

In summary, there is consistent evidence that desexing is associated with an increased risk of obesity in dogs of both sexes across a wide variety of environments, as well as some evidence that weight loss is more difficult to achieve in desexed as compared to intact dogs, and that caloric intake is decreased by the presence of sex steroids at least in female dogs.

#### 5.2.7. Other Disease Risks

Various other disease risk increases associated with desexing in one or both sexes have been published for both the overall dog population and in studies limited to specific breeds:

A retrospective study based on n = 90,090 patient records from a US veterinary teaching hospital found that gonadectomized dogs of both sexes were at a significantly higher risk of immune-mediated diseases including atopic dermatitis, autoimmune hemolytic anemia, Addison’s disease, hypothyroidism, immune-mediated thrombocytopenia, and inflammatory bowel disease, with the effect being stronger in females compared to males except for autoimmune hemolytic anemia and Addison’s disease [195]. In this context, a study on Golden and Labrador Retrievers found that desexed dogs had lower IgE levels than intact dogs in both sexes; however, this difference was not associated with clinical disease [115].

Another retrospective study based on the same veterinary teaching hospital patient records found that gonadectomized dogs of both sexes were less likely to be diagnosed with several congenital or early-onset inherited disorders including aortic stenosis, early onset cataracts, patent ductus arteriosus, ventricular septal defect, and portosystemic shunt. The same study also found that gonadectomized males were less likely to be diagnosed with gastric dilation volvulus and dilated cardiomyopathy as compared to intact males, and that gonadectomized dogs of both sexes had an increased risk of hyperadrenocorticism and epilepsy [162]. In this context, it is likely that the reduced risk of congenital or early-onset inherited disease reflects a selection bias within the gonadectomized population as opposed to an increased baseline risk.

As mentioned under the previous point, diabetes mellitus has been reported to be more common in desexed male, but not female dogs compared to intact dogs across the whole dog population [193]. 

Finally, one study of dogs affected with idiopathic epilepsy based on the VetCompass database using Labrador Retriever and Border Collie patient records from private veterinary practices in the UK found that 74% of gonadectomized patients were gonadectomized before the onset of seizures, and that dogs that had been gonadectomized before the onset of seizures had significantly longer survival times than those that had been gonadectomized afterwards [119]. 

In summary, a wide variety of additional disease risk increases and decreases has been reported in the literature; however, most of these findings have not thus far been independently reproduced.

## 6. Behavior

The desexing methods that result in the elimination of sex steroids have been shown to affect behavior in multiple studies. As is the case in the literature on other aspects of desexing, most studies are specific to surgical gonadectomy; however, there are also a few studies that have examined the effects of non-surgical methods, most prominently those of GnRH superagonist implants and progestagen administration.

Review articles exist on the topic of desexing effects on canine behavior for both the effects of gonadectomy [5,196] and GnRH superagonist treatment [197]. Most studies on behavioral changes associated with desexing published thus far have been specific to male dogs, while comparably few of them have also included or focused on behavior in gonadectomized female dogs. In studies that differentiated between effects in males and females, the behavioral effects of desexing were generally found to be more pronounced in males than they were in females [190].

An important clinical issue in desexing and behavior is presented by the difficulty to predict which desirable or undesirable behavioral changes will occur in which individual dogs [198], which is particularly concerning given the irreversible nature of gonadectomy. In this context, we note that in one study based on companion dog owner surveys in Australia, 61% of male dog owners and 47% of female dog owners stated that they would not proceed with desexing their dog if they were to be given the same choice again [199]. Where available, fully reversible desexing methods such as GnRH superagonist implants are therefore a particularly valuable tool in situations where desexing is considered as a behavioral intervention, as treatment can be discontinued and the hormonal *status quo ante* restored in case of undesirable outcomes [197].

Based on the existing literature, the effects of desexing on behavior can be divided into libido and its associated behaviors such as roaming and certain aspects of mounting, urinary marking, dog bite injury risk in humans and conspecifics, various other forms of boldness-related, aggressive or reactive behaviors, and cognitive function.

### 6.1. Libido and Associated Behaviors

Testosterone controls libido and attraction to estrous females, which first appear in intact male dogs between ages 4 and 6 months [200]. Sexual mounting and copulatory behavior is mediated by testosterone effects on the medial preoptic-anterior hypothalamus [201]. While desexing of sexually inexperienced dogs generally eliminates libido and copulatory behavior, attraction to female dogs including copulatory behavior may persist in sexually experienced male dogs even after desexing [202]. GnRH superagonist implants initially stimulate the pituitary-gonadal axis, which may result in a transient increase in libido and associated behaviors following the initiation of treatment [197].

Several studies have found that gonadectomy significantly reduces mounting of conspecifics and roaming behavior in 60% to 90% of male companion dogs [191,203,204]. However, one study found increased mounting of objects in desexed males [205], and in a study on free-roaming dogs, neither roaming nor mounting behavior was reduced in free-roaming dogs undergoing either surgical or hormonal desexing when compared to their intact conspecifics [206]. Various studies have found no effect of age at desexing on its effects on either roaming or mounting behavior [5,198,203]. In this context, it is important to note that mounting behavior can also occur for reasons not related to libido, such as stress or anxiety, and that this would most likely contribute to the observed lack of effect of desexing on mounting behavior in some cases [207].

In summary, and unsurprisingly, the literature consistently shows that desexing reduces libido and its associated behaviors such as conspecific mounting and roaming in the majority of desexed male companion dogs. Sexual behavior persistence after desexing appears to depend on previous sexual experience rather than age at desexing. Interestingly, desexing effects on conspecific mounting and roaming behaviors do not appear to be present in free-roaming dogs. 

### 6.2. Urinary Marking

Inappropriate urinary marking in male companion dogs is a behavior that may cause considerable distress to their owners and accounts for a significant percentage of dogs being relinquished to shelters [208,209]. Male-pattern urinary marking is a testosterone-dependent behavior that is initiated during puberty [210,211], but which contrary to mounting and copulatory behavior does not depend on testosterone effects in the preoptic-anterior hypothalamus [201]. The majority of studies of desexing found a significant decrease in urinary marking behavior in desexed male dogs regardless of age at desexing [5,190,191,203,204], while one study found an influence of age at desexing, with later ages being associated with a less pronounced decrease in marking following desexing [205]. In contrast, female dogs do not generally show urinary marking, and urinary behavior is not generally affected by desexing in female dogs [212].

In summary, there is consistent evidence across studies that desexing reduces the frequency of urinary marking in the majority of male dogs, and mostly consistent evidence that this effect does not depend on the age at desexing.

### 6.3. Bite Injury Risk

Dog bite injury risk is an aspect of dog behavior that has substantial societal and public health implications. Dog bites cause an estimated 580,000 human injuries and an average of 20 human deaths in the US per year, with 51% of reported dog bite cases occurring in children under age 12 [213]. Consequently, a considerable body of literature based on dog bite injury report data exists, some of which also includes the effects of desexing [214,215,216,217]. 

A systematic literature review based on observational studies of dog bite risk concluded that five out of six of the articles considered showed that intact dogs were more likely than desexed dogs to cause bite injuries; however, the authors also concluded that the available data was insufficient to estimate the effect size, and that all studies considered were observational case-control studies rather than interventional studies [196]. All of these studies were based on dogs from the US and/or Canada, and in this context, as is the case for health outcomes, it should be considered that desexing may act as a proxy for overall better animal husbandry standards in these populations. 

Dog bite risk has also been studied in free-roaming dog populations. In one such study, a street dog desexing program in Jaipur, India resulted in a decrease of human bite injuries that the authors attributed to a decrease in female dogs showing maternal protection behavior [96].

In summary, there is mostly consistent evidence for a reduction in human bite injury risk associated with desexed dogs; however, in the companion dog population this is confounded by the fact that all studies thus far have been performed in populations where desexing acts as a proxy for overall better animal husbandry. In practice, a thorough behavioral evaluation of any dog with a bite history is paramount to identify the underlying motivation for the bite incident [218].

### 6.4. Boldness-Related and Aggressive Behavior

The effect of desexing on boldness-related and aggressive behavior has also been considered in a variety of studies based on owner questionnaires and surveys using both the overall dog population and specific breeds. Boldness is defined as one end of the shyness–boldness axis in dog personality that incorporates several different personality traits such as playfulness, sociability including fear of or aggression towards people, and curiosity/fearlessness [219,220,221]. It can be reproducibly measured in standardized owner questionnaires such as C-BARQ [222]. Aggression is a trait within the boldness spectrum that has been fairly extensively studied in dogs, and is generally divided into aggression directed at humans and aggression directed at other dogs. 

One study on n = 1054 companion dogs in Australia using the C-BARQ questionnaire found that male dogs scored higher in boldness traits than female dogs, and that desexed dogs of both sexes scored significantly lower in boldness traits than intact dogs [223]. On the other end of the shyness-boldness spectrum, several studies have reported an increase in fearfulness and/or reactivity associated with gonadectomy. This included increased fearfulness in gonadectomized female Labrador Retrievers [212] and gonadectomized Vizslas of both sexes [112], as well as reactivity and vocalization in ovariectomized German Shepherd females [224,225]. 

A study of n = 13,796 dog owners using the C-BARQ questionnaire found no effect of gonadectomy on aggression towards familiar people or dogs across both sexes, but found a small but significant increase in aggression towards strangers in dogs that had been gonadectomized between ages 7 and 12 months [226]. A different study also using the C-BARQ questionnaire and focusing on n = 6235 male dogs that had been orchiectomized for non-behavioral reasons before age 10 years found that a longer percentage of lifespan spent desexed was associated with both increased fearfulness and increased aggression [205].

Two studies based on owner questionnaires of n = 2207 dogs found that desexed males were significantly more likely to show both dog-directed and human-directed resource guarding aggression than all other demographics [227,228].

Several studies have investigated various forms of aggression in male dogs that were orchiectomized for behavioral reasons using owner interviews or questionnaires. One such study of 57 dogs found that aggression towards family members was reduced in 30% of dogs, while aggression towards strangers or intruders was reduced in 10% of dogs following orchiectomy [198]. In contrast, similar a study of n = 122 orchiectomized male dogs found that inter-male dog aggression was reduced in about 60% of dogs following orchiectomy [191]. A similar retrospective study of n = 42 orchiectomized male dogs found reduced aggression towards other male dogs in 60% of dogs, but no reduction in territorial or fear-induced aggression [203].

Finally, an observational study on n = 93 Hungarian dogs that measured both obedience and owner-directed aggression found that males and intact dogs tended to be less obedient than females and gonadectomized dogs, but that gonadectomized dogs that were less obedient were also more likely to show signs of owner-directed aggression [229].

In summary, the evidence for an influence of desexing on boldness-related and aggressive behavior is inconsistent and sometimes contradictory. The most consistent finding appears to be a reduction in dog-directed aggression in male dogs that have been orchiectomized due to behavioral problems; however, this conclusion is based on a comparably low number of dogs and may not apply to males without pre-existing behavioral issues. Similarly, in studies that did not include information on the owners’ motivation for desexing, there may have been a selection of dogs that were showing problematic behaviors before desexing, which also raises issues regarding the potential of misdiagnosis in case a thorough behavioral assessment was not carried out. In addition, many of the available studies do not differentiate between males and females when considering behavioral outcomes of desexing; however, considering the important differences in endocrine physiology between the sexes, it would not be surprising to find that effects of desexing on boldness-related and aggressive behavior could differ between the sexes.

### 6.5. Cognitive Function

Some literature exists on the impact of gonadectomy on cognitive function in dogs, most notably regarding Canine Cognitive Dysfunction (CCD), which is a behavioral syndrome affecting older dogs that shares many pathophysiological and behavioral hallmarks with human Alzheimer’s Disease (AD), including progressive cognitive impairment, loss of normal sleep patterns, increased anxiety, and aimless wandering as well as Amyloid-beta and possibly tau pathology in the brain [230,231,232]. In this context, female and gonadectomized dogs have been shown to be significantly more likely to show signs of CCD than intact and male dogs in a study of n = 325 geriatric dogs aged nine or older [233]. CCD has also been shown to progress more rapidly in gonadectomized male dogs that already have signs of mild cognitive impairment compared to intact males with mild cognitive impairment during a 12–18 month follow-up period [234]. Interestingly, this mirrors findings in human patients, where women have an increased AD risk as compared to men [235], and where men receiving androgen deprivation therapy for prostate cancer have been shown to be at a higher risk of developing dementia and AD than controls [236].

Apart from CCD, there is some limited evidence from standardized behavioral tests on companion dogs that found cognitive differences related to desexing. In one such study on n = 64 dogs that were grouped by sex and gonadectomy status and tested in a spatial learning maze task, the authors found that intact females had the highest success rates of all four groups in both learning and completing the task, as well as memorizing it after a latency period of two weeks, while there was no difference between ovariectomized females, intact and orchiectomized males in either outcome [237]. Interestingly, female, but not male dogs have also been shown to react to a size consistency violation in an object display test using 25 male and 25 female companion dogs; however, there was no difference between orchiectomized and intact males [238].

In summary, there is some limited evidence that gonadectomy increases the risk of cognitive dysfunction in both sexes, and increases the speed of progression from mild to more severe cognitive impairment in male dogs, which interestingly seems to mirror sex-specific aspects of Alzheimer’s Disease in humans. There is also evidence for differences in spatial learning between male and female dogs that appear to be influenced by ovariectomy, but not orchiectomy; however, the field is very much in its infancy and more research is needed to further characterize these differences and how they are influenced by desexing.

## 7. Conclusions

While a wide variety of surgical and non-surgical desexing methods have been described in the literature, in practice gonadectomy is by far the most commonly used desexing method, and the great majority of studies on desexing have been limited to gonadectomy as opposed to other forms of desexing. Desexing rates are highly variable depending on geographic area even within the Western world. The terminology used to describe various desexing methods is inconsistent and contradictory, and there appears to be a need for the adaption of standardized terminology in the literature.

is the available empirical evidence shows a clear lack of an effect of desexing as a population control measure in companion and shelter dogs, which is surprising given that desexing is commonly recommended in the literature to achieve this goal. However, there is evidence that desexing is effective for population control in free-roaming dog populations and may have unrelated health benefits in these populations by reducing bite risk and risk of zoonotic disease transmission due to combined desexing and vaccination efforts. Mathematical modelling studies indicate that most of the population control effect in these populations can be attributed to female rather than male desexing.

A large amount of literature related to disease risk increases and decreases associated with desexing exists, but commonly neglects to consider the effect of age as the most important risk factor for most of the studied diseases. The largest effects on common and commonly life-limiting diseases appear to be seen in the female reproductive tract, most notably with regards to pyometra and, to a lesser extent, mammary tumors. While some disease risks appear to be consistently affected by desexing across both sexes and all breeds, other risk changes appear to be sex- and/or breed-specific, and more research is needed to determine the extent of variation between breeds and sexes for these diseases. Disease risks that are consistently reported to be increased with desexing in both sexes and across breeds include cranial crucial ligament rupture, hemangiosarcoma, lymphoma, transitional cell carcinoma of the bladder, and obesity.

In contrast, there are relatively few studies on the effects of desexing on lifespan in dogs, and the available studies are subject to multiple biases associated with desexing being a proxy for overall better husbandry and veterinary care, desexed dogs already having lived to the age at which the procedure was performed, and right censored data in case of studies based on death records. However, when taken together, there is consistent evidence for a beneficial effect of desexing on lifespan in female dogs, and inconsistent evidence for such an effect in male dogs, and the male-specific effect is consistently shown to be of lower magnitude than the female-specific effect if present.

The amount of literature on the behavioral outcomes associated with desexing is larger for male than for female dogs and consistently shows a reduction in libido, roaming, conspecific mounting, and urinary marking frequency in a large percentage of orchiectomized males, as well as a reduction in inter-male dog aggression in male dogs that were orchiectomized because of existing behavioral problems. Limited evidence also exists for desexing increasing the risk and progression of age-related cognitive dysfunction. In addition, there is some evidence that desexed dogs of both sexes are less likely to cause bite injuries to humans; however, these studies have been conducted in environments where desexing is expected to act as a proxy for overall better husbandry.

The evidence for other behavioral effects such as human-directed aggression, human or object mounting, resource guarding, shyness and anxiety-related behaviors is inconsistent and sometimes contradictory, and often fails to differentiate between the sexes. There are relatively few studies specific to females or individual breeds, and more research is needed to further elucidate the influence of desexing in these populations.

One additional limitation of the available literature on the various effects of desexing is that most of it reflects post hoc observational studies, not randomized interventional studies. As such, most of the effects described in the literature are necessarily correlations that do not strictly establish causality with desexing. Where feasible, more research is needed using prospective study designs.

In conclusion, we find that the evidence for an overall beneficial effect of desexing is stronger in female than in male dogs when considering both population control and health benefit aspects; however, the available evidence suggests that there is significant variation between the breeds and sexes, and more research is needed to further elucidate these differences and to arrive at more individualized evidence-based recommendations for clinical practice.
